# Direct Effects of 308-nm Excimer Light on Keratinocyte Proliferation and Differentiation in Human Organ-Cultured Skin

**DOI:** 10.3390/ijms27156698

**Published:** 2026-07-27

**Authors:** Ayaka Okazaki, Hisayoshi Imanishi, Hiroyuki Kunimoto, Daisuke Tsuruta

**Affiliations:** 1 Department of Dermatology, Graduate School of Medicine, Osaka City University, Osaka 5458585, Japan; 2 Department of Dermatology, Graduate School of Medicine, Osaka Metropolitan University, Osaka 5458585, Japan; 3 Department of Immunology, Graduate School of Medicine, Osaka Metropolitan University, Osaka 5458585, Japan

**Keywords:** 308-nm excimer light, keratinocyte, human organ-cultured skin, proliferation, apoptosis, RNA sequencing, MAPK signaling pathway

## Abstract

Targeted phototherapy using 308-nm excimer light (EL-308) is widely employed to treat localized skin diseases, such as psoriasis and vitiligo. Although its immunomodulatory effects are well established, tissue-level epidermal responses to EL-308 in human skin explants have not been fully characterized in a controlled ex vivo setting. Therefore, the present study examined dose- and time-dependent changes in keratinocyte proliferation, apoptosis, and differentiation markers in a human organ-cultured skin model without systemic immune inputs (i.e., without circulation-dependent immune-cell recruitment). Human organ-cultured skin samples were irradiated with EL-308 at 300, 400, or 600 mJ/cm^2^ and analyzed on days 2, 5, and 7 using immunohistochemical markers, including Ki-67, TUNEL, CK10, CK16, involucrin, and integrin β1. RNA sequencing was performed 24 h after irradiation with 600 mJ/cm^2^, followed by KEGG and GO enrichment analyses. EL-308 exposure was associated with dose- and time-dependent changes in Ki-67 and TUNEL labeling and with altered expression of differentiation-related markers (integrin β1, CK16, CK10, and involucrin). Transcriptomic analyses revealed enrichment of metabolic and epidermis-related gene sets, including keratinization and epidermal development terms, consistent with a tissue-level response to UVB exposure. These findings provide descriptive data on epidermal marker dynamics and associated transcriptional programs after EL-308 irradiation in human organ-cultured skin and may inform future studies designed to evaluate early damage markers and functional outcomes.

## 1. Introduction

Phototherapy has played a central role in the treatment of various dermatological conditions, including psoriasis, vitiligo, cutaneous T-cell lymphoma, mycosis fungoides, alopecia areata, atopic dermatitis, and pruritus [1]. Multiple mechanisms are involved in its therapeutic benefits, such as the induction of apoptosis in epidermal T lymphocytes and keratinocytes (KCs), the modulation of cytokine expression, and general immunosuppression [2,3,4]. Phototherapy, with the exception of PUVA, is generally considered to be safe, cost-effective, and appropriate for use in vulnerable populations, such as children, the elderly, and pregnant women, particularly when compared to systemic therapies.

A major challenge in phototherapy is reducing the frequency and extent of irradiation to minimize long-term adverse effects, such as photoaging and skin carcinogenesis [5].

To address this issue, targeted narrow-band UVB (NB-UVB) therapies, such as the 308-nm excimer light (EL-308), have been developed to deliver high-intensity treatment selectively to affected areas while sparing surrounding healthy tissue [6].

This precision is achieved through the unique emission characteristic of xenon chloride excimer lamps, which generate monochromatic light with a wavelength of 308 nm, enabling focused delivery with minimal systemic exposure [6,7,8]. EL-308 enables high-fluence, targeted treatment without damage to surrounding skin and has demonstrated clinical efficacy for localized inflammatory skin conditions, such as vitiligo and psoriasis [9]. It works by inducing apoptosis in pathogenic T cells, enhancing regulatory T-cell function, up-regulating the expression of immunoregulatory cytokines, such as interleukin-10, and promoting cell cycle arrest in KCs via the activation of p53 and down-regulation of Bcl-2 [7,10,11]. Despite this progress, the majority of research conducted to date has yet to show its direct effects on epidermal KCs at the tissue level. Recent meta-analyses support its clinical efficacy; however, the underlying mechanisms at the KC level remain unclear [12,13,14].

A recent study reported that 308-nm UVB irradiation can modulate epidermal repair-associated responses and wound closure in experimental settings, together with changes in signaling pathways relevant to tissue repair [15]. However, direct epidermal responses to EL-308 at the human tissue level, separated from systemic immune influences, remain incompletely defined. Here, using a human ex vivo organ-cultured skin model to eliminate systemic immune inputs, we aimed to characterize early-to-late, dose-dependent keratinocyte phenotypes (proliferation, apoptosis, differentiation) and to profile early transcriptional programs after EL-308 exposure. The goal of the present study was not to fully elucidate the molecular mechanisms of EL-308. To the best of our knowledge, no other studies have used this model to examine the effects of EL-308 on human skin tissue.

## 2. Results

### 2.1. EL-308 Irradiation Is Associated with Increased Keratinocyte Proliferation on Day 2

**Figure 1:**
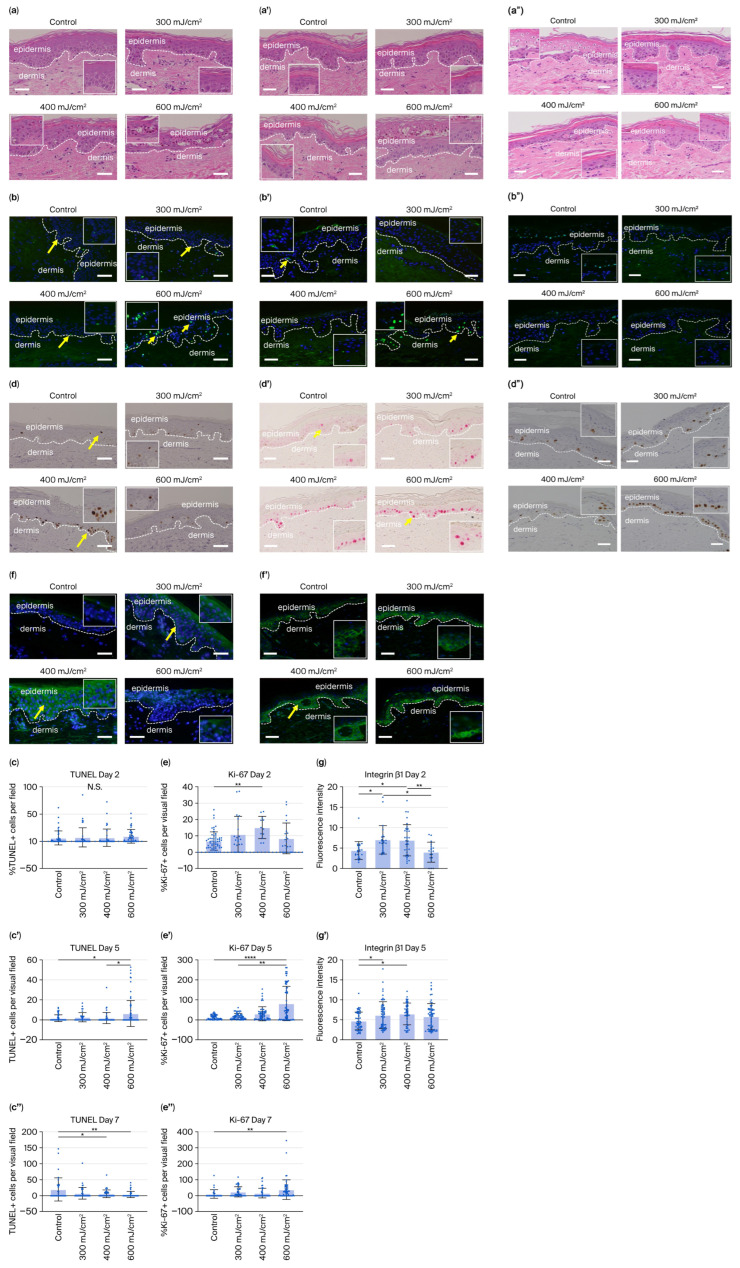
Effects of EL-308 on keratinocyte proliferation, apoptosis, and stem cell marker expression on day 2, day 5, and day 7. Human organ-cultured skin samples were irradiated with 300, 400, or 600 mJ/cm^2^ of EL-308 and collected on day 2 (**a**–**g**), day 5 (**a’**–**g’**), and day 7 (**a”**–**e”**). (**a**,**a’**,**a”**) H&E staining revealed dose-dependent morphological changes, with 600 mJ/cm^2^ inducing epidermal alterations. (**b**,**b’**,**b”**) Representative TUNEL staining images. (**c**,**c’**,**c”**) Quantification showed increased apoptotic nuclei at 600 mJ/cm^2^ on day 5. (**d**,**d’**,**d”**) Representative Ki-67 immunostaining images. (**e**,**e’**,**e”**) Ki-67 positive nuclei were significantly increased in the 400 and 600 mJ/cm^2^ groups. (**f**,**f’**) Representative integrin β1 immunofluorescence staining. (**g**,**g’**) Integrin β1 fluorescence intensity was elevated at 300 and 400 mJ/cm^2^. Inset images highlight the corresponding high-magnification regions outlined by white boxes, allowing visualization of detailed staining patterns. Yellow arrows indicate representative areas of marker expression, and dashed lines indicate the epidermal-dermal junction. For each donor, multiple non-overlapping microscopic fields within the viable epidermis were examined at ×400 magnification. Each dot in the graphs represents one microscopic field. Three independent donors were included in the study (*n* = 3). The viable epidermis was manually outlined as the region of interest. TUNEL- and Ki-67-positive nuclei were quantified as the number of positive cells per epidermal area, and integrin β1 expression was measured as the mean gray value in ImageJ with standard background subtraction. All images were acquired under identical exposure settings. Data are presented as the mean ± SD calculated from three independent donors (*n* = 3). Statistical analyses were performed using one-way ANOVA followed by Tukey’s multiple comparisons test. N.S., not significant; * *p* < 0.05, ** *p* < 0.01, **** *p* < 0.0001. Scale bar = 50 μm.

To examine the direct effects of EL-308 on epidermal KCs, we employed a human organ-cultured skin model and irradiated samples with 300, 400, or 600 mJ/cm^2^ of EL-308 (Thera Beam UV308; USHIO, Tokyo, Japan). The 300 mJ/cm^2^ dose was selected based on previously reported minimal erythema dose thresholds for human skin [16]. We also wanted to investigate the effects of EL-308 at higher doses; 400 and 600 mJ/cm^2^ were selected. A histological analysis on day 2 revealed no notable morphological changes in the epidermis following irradiation with 300 or 400 mJ/cm^2^, whereas 600 mJ/cm^2^ induced visible tissue damage. This dose-dependent injury pattern is consistent with previous histological assessments following UVB exposure (Figure 1a) [17].

Terminal deoxynucleotidyl transferase-mediated dUTP nick-end labeling (TUNEL) staining was performed to assess apoptosis. The number of TUNEL-positive cells in the epidermis did not significantly differ among irradiation groups at this time point (Figure 1b,c). In contrast, immunohistochemical staining for Ki-67 showed that the number of proliferating KCs was significantly higher after the 400 mJ/cm^2^ treatment than in control samples (Figure 1d,e).

Since epidermal stem/progenitor cells contribute to tissue maintenance and repair [18,19,20,21], we also assessed integrin β1 expression, a marker enriched in basal keratinocytes. Recent reviews emphasized its role in epidermal adhesion and stem cell-associated compartments [22]. Irradiation with both 300 and 400 mJ/cm^2^ led to higher integrin β1 expression than in control and 600 mJ/cm^2^-irradiated samples (Figure 1f,g). Together with the Ki-67 findings, these results indicate that, at sub-damaging doses, EL-308 exposure was associated with increased proliferative labeling and higher integrin β1 signal during the early post-irradiation phase in this ex vivo model.

### 2.2. EL-308 Irradiation Is Associated with Increased Apoptosis and Proliferation on Day 5

To investigate responses to EL-308 irradiation beyond the early period, we examined epidermal samples on day 5 post-treatment. The histological evaluation showed partial restoration of epidermal architecture in the group exposed to 600 mJ/cm^2^, whereas minimal changes were noted at lower doses (Figure 1a’). These structural changes are consistent with prior reports describing re-epithelialization-like processes in ex vivo human skin models after UVB exposure [23].

TUNEL assays revealed a significant increase in apoptotic keratinocytes in the 600 mJ/cm^2^ group, suggesting dose-dependent cytotoxicity (Figure 1b’,c’). This result is consistent with previous findings showing UVB-induced keratinocyte apoptosis in human skin explants and culture models [24]. Ki-67 immunostaining conducted simultaneously showed a marked increase in proliferative labeling within the epidermis, consistent with a concurrent compensatory proliferative response (Figure 1d’,e’).

To evaluate whether integrin β1 expression changed over time, we analyzed integrin β1 staining on day 5. Elevated integrin β1 levels were observed in the 300 and 400 mJ/cm^2^ group (Figure 1f’,g’). Overall, these results indicate dose- and time-dependent epidermal responses after EL-308 exposure, including concurrent increases in apoptosis and proliferation markers after high-dose irradiation.

### 2.3. EL-308 Irradiation Is Associated with Altered Proliferation and Apoptosis on Day 7

To evaluate later responses to EL-308, epidermal samples were examined on day 7 after irradiation. Control tissues showed marked degenerative changes, which are consistent with the known limitations of extended human skin organ culture under unstimulated conditions. In comparison, EL-308-treated samples, particularly those exposed to 600 mJ/cm^2^, appeared to retain a more preserved epidermal architecture (Figure 1a”).

TUNEL assays showed lower numbers of apoptotic nuclei in both the 400 and 600 mJ/cm^2^ groups than in control samples (Figure 1b”,c”). Concurrently, Ki-67 staining revealed higher numbers of Ki-67-positive nuclei in the 600 mJ/cm^2^ group (Figure 1d”,e”). These findings suggest that EL-308 exposure can be followed by changes in the balance of apoptotic and proliferative labeling at later time points under organ-culture conditions; however, interpretation is limited by the ex vivo setting and baseline degeneration in control tissues.

Taken together, the day-7 data indicate that EL-308 exposure was associated with altered proliferation and apoptosis markers in the epidermis during organ culture. Whether these marker changes reflect functional repair, persistent stress responses, or culture-dependent effects requires further validation in designs that include early time points, DNA-damage assessment, and in vivo models.

### 2.4. EL-308 Modulates the Expression of Cytokeratin 10 (CK10), Involucrin, and Cytokeratin 16 (CK16) in a Dose-Dependent Manner

To investigate the impact of EL-308 on KC differentiation, we assessed the expression of CK10, involucrin, and CK16, which are key markers associated with KC maturation and epidermal turnover.

**Figure 2:**
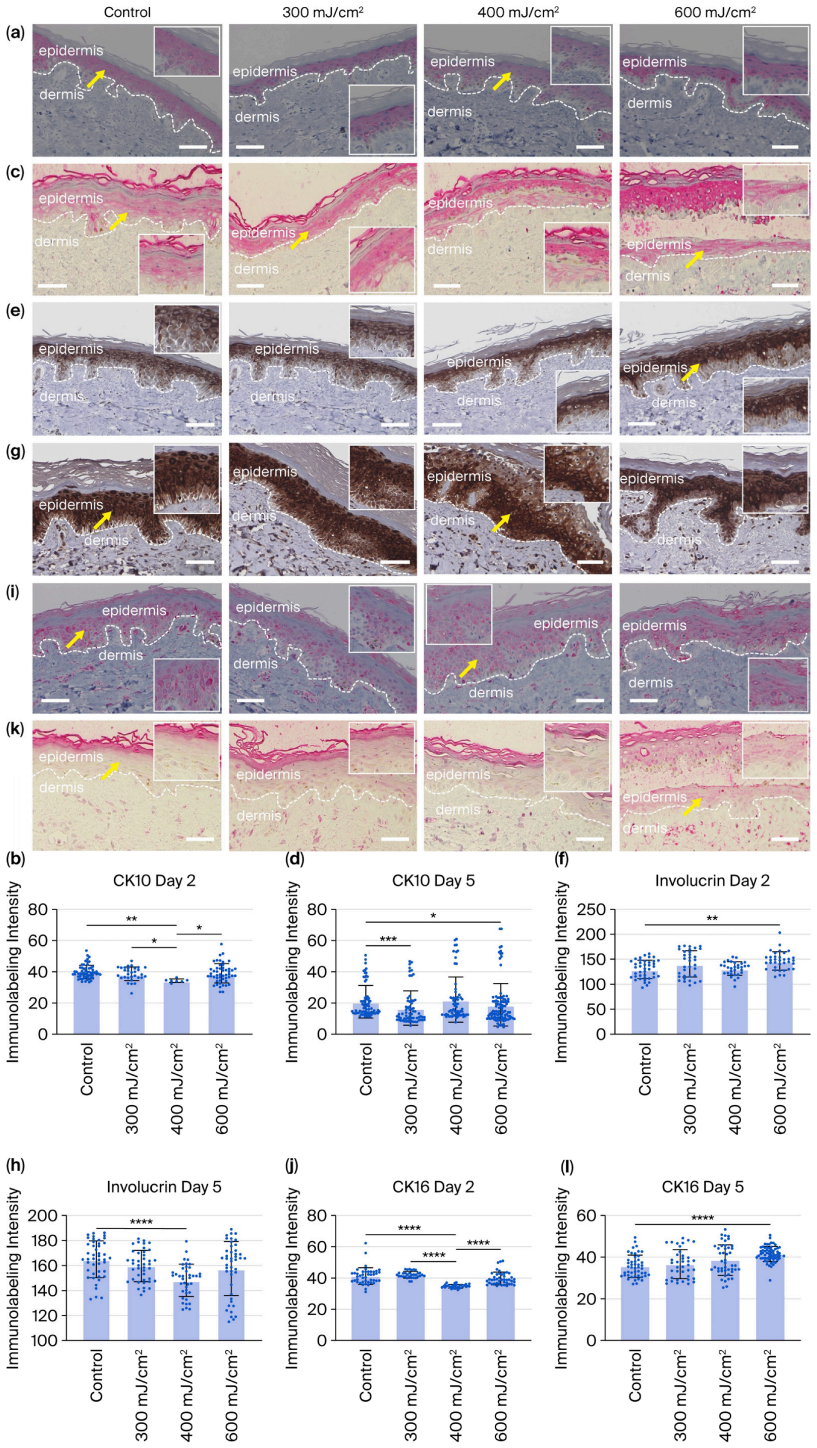
Effects of EL-308 on keratinocyte differentiation markers on day 2 and day 5. (**a**,**b**) CK10 expression was significantly decreased at 400 mJ/cm^2^ on day 2. (**c**,**d**) CK10 expression was reduced at 300 and 600 mJ/cm^2^ on day 5. (**e**,**f**) Involucrin expression was increased at 600 mJ/cm^2^ on day 2. (**g**,**h**) Involucrin expression was decreased at 400 mJ/cm^2^ on day 5. (**i**,**j**) CK16 expression was significantly decreased at 400 mJ/cm^2^ on day 2. (**k**,**l**) CK16 expression was significantly elevated at 600 mJ/cm^2^ on day 5. Inset images highlight the corresponding high-magnification regions outlined by white boxes, allowing visualization of detailed cytoplasmic staining patterns. Yellow arrows indicate representative areas of marker expression, and dashed lines indicate the epidermal-dermal junction. For each donor, multiple non-overlapping microscopic fields within the viable epidermis were examined at ×400 magnification. Each dot in the graphs represents one microscopic field. Three independent donors were included in the study (*n* = 3). The viable epidermis was manually outlined as the region of interest. CK10, involucrin, and CK16 signals were quantified as the mean gray value within the epidermal region using ImageJ with standard background subtraction. All images were acquired under identical exposure settings. Data are presented as the mean ± SD calculated from three independent donors (*n* = 3). Statistical analyses were performed using one-way ANOVA followed by Tukey’s multiple comparisons test. * *p* < 0.05, ** *p* < 0.01, *** *p* < 0.001, **** *p* < 0.0001. Scale bar = 50 μm.

CK10, a well-established marker of KC terminal differentiation normally expressed in the spinous and granular layers of the epidermis [25], was significantly reduced on day 2 after 400 mJ/cm^2^ irradiation. This result aligns with previous findings showing the UVB-induced down-regulation of CK10 expression in a stressed epidermis. On day 5, CK10 expression was reduced in the 300 and 600 mJ/cm^2^ groups (Figure 2a–d). These results may reflect delayed differentiation or epidermal damage.

Involucrin, another marker of terminal differentiation involved in forming the cornified envelope [26], exhibited a biphasic response. Stress-induced up-regulation of involucrin has previously been reported in vitro [27]. Its expression increased at 600 mJ/cm^2^ on day 2 but significantly decreased at 400 mJ/cm^2^ on day 5 (Figure 2e–h). This pattern may reflect a transient stress-associated change followed by altered differentiation during the subsequent culture period.

In contrast, the expression of CK16, which is associated with keratinocyte activation and rapid turnover [28,29], was significantly downregulated in the 400 mJ/cm^2^ group on day 2. On day 5, CK16 expression was significantly up-regulated in the 600 mJ/cm^2^ group compared to the control (Figure 2i–l). These results suggest that CK16 expression changed in a dose- and time-dependent manner, consistent with a stress-associated epidermal response to UVB exposure in this ex vivo model.

Collectively, these results demonstrate dose- and time-dependent changes in differentiation-related markers after EL-308 exposure. The functional implications of these marker changes (e.g., whether they reflect adaptive repair or persistent stress) cannot be determined from the present ex vivo data alone and warrant further investigation. A summary of these immunohistochemical findings is provided in Supplementary Table S2.

### 2.5. Transcriptomic Changes Induced by EL-308: Metabolic Reprogramming and Epidermis-Related Transcriptional Programs

To profile early transcriptional responses at the tissue level underlying the histological changes induced by EL-308, we performed RNA sequencing on organ-cultured human skin samples 24 h after exposure to 600 mJ/cm^2^ irradiation. A transcriptomic analysis revealed marked differences in gene expression compared with non-irradiated controls (Supplementary Table S1).

**Figure 3:**
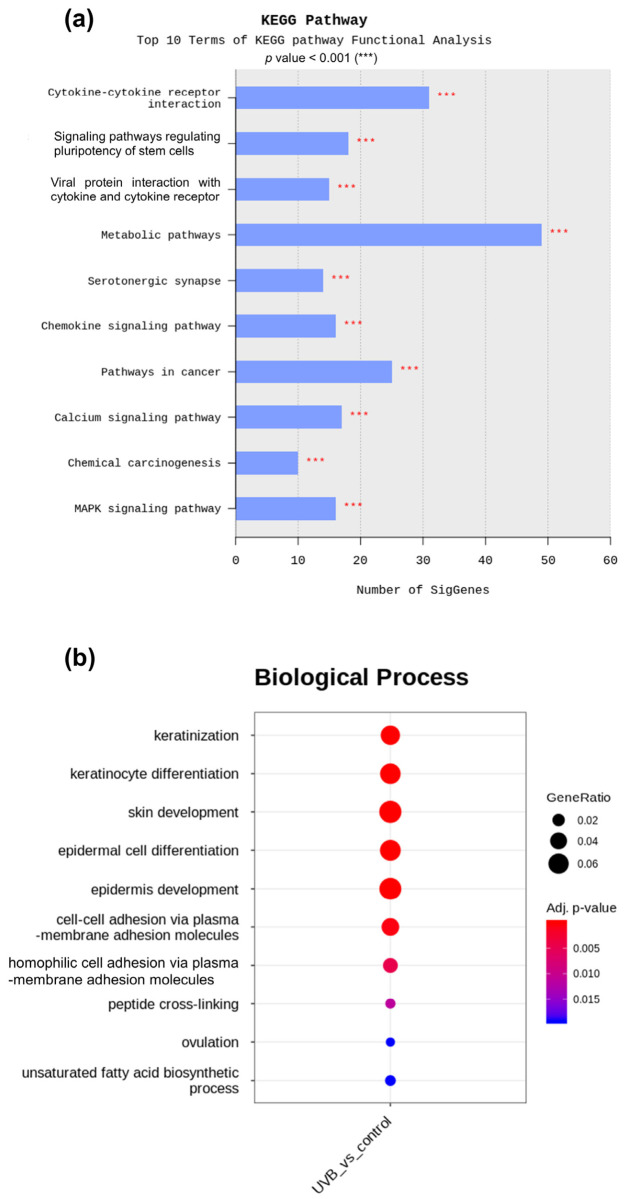
Transcriptomic changes following EL-308 irradiation. (**a**) A KEGG pathway analysis revealed enrichment in metabolic pathways, MAPK signaling, cytokine–cytokine receptor interactions, and chemokine signaling. (**b**) A GO analysis showed enrichment in keratinization, keratinocyte differentiation, and epidermis development.

The Kyoto Encyclopedia of Genes and Genomes (KEGG) pathway enrichment analysis demonstrated significant up-regulation of genes involved in metabolic pathways (adjusted *p* < 0.001), followed by enrichment in MAPK signaling, cytokine–cytokine receptor interaction, and chemokine signaling pathways (Figure 3a; Supplementary Figure S1). These pathways are broadly involved in cellular stress responses and epidermal biology.

In agreement with these findings, a gene ontology (GO) analysis showed significant enrichment in biological processes related to keratinization, keratinocyte differentiation, and epidermis development (Figure 3b), indicating the presence of epidermis-related transcriptional programs after EL-308 exposure.

To further examine functional targets, we focused on genes within the MAPK and PI3K-Akt signaling pathways, which are key regulators of epidermal homeostasis and also mediate keratinocyte survival and mitogenic responses [30]. We noted changes in upstream ligands (e.g., FGF2, FGF7, and IGF1) and downstream effectors (e.g., ATF2, MAP4K3, RPS6KA6, and PIK3CA), with fold changes ranging from 3.1 to 6.8 (Supplementary Figure S2). Although these differences did not reach statistical significance after multiple testing correction (adjusted *p* ≥ 0.05), their upward trends may suggest a possible supportive role in EL-308-associated keratinocyte activation. Accordingly, these data are presented as supplementary information and should not be interpreted as definitive pathway activation.

Collectively, our transcriptomic findings are consistent with tissue-level metabolic changes and altered differentiation-related programs in human epidermis after EL-308 exposure under organ-culture conditions.

## 3. Discussion

An evaluation of the biological effects of EL-308 was conducted herein using a human organ-culture skin model. In this exploratory ex vivo study, our goal was not to fully elucidate the molecular mechanisms of EL-308, but rather to describe dose- and time-dependent epidermal phenotypes and associated tissue-level transcriptional responses in an ex vivo organ-cultured skin model without systemic immune inputs. By integrating histological, immunohistochemical, and transcriptomic analyses, we observed that EL-308 exposure was associated with changes in proliferation, apoptosis, differentiation markers, and metabolic gene sets. These results provide descriptive tissue-level data that may help interpret epidermal responses to targeted excimer phototherapy, while emphasizing the need for careful consideration of UVB-induced injury and repair dynamics.

Histological and immunohistochemical analyses revealed dose- and time-dependent responses to EL-308. At lower doses, integrin β1 staining was higher than in controls. At the higher dose (600 mJ/cm^2^), the number of TUNEL-positive cells increased on day 5, consistent with apoptotic clearance of UVB-damaged keratinocytes. The coexistence of apoptotic and proliferative labeling on day 5 is compatible with a damage–response program in which apoptotic clearance and compensatory proliferation can co-occur after UVB exposure.

Furthermore, EL-308 affected epidermal differentiation markers. The expression of CK10 and involucrin, both markers of terminal differentiation, was generally reduced at later time points, although involucrin showed a transient increase at high doses on day 2. In contrast, CK16, a stress-associated keratin [31], showed a biphasic pattern (decreased at 400 mJ/cm^2^ on day 2 and increased at 600 mJ/cm^2^ on day 5). These observations are consistent with dose-dependent stress responses in the epidermis after UVB exposure; however, we did not directly measure DNA damage or signaling intermediates, and mechanistic interpretations should be considered hypothesis-generating.

The transcriptomic analysis provided additional context for these phenotypic findings. While immediate signaling responses to UV exposure can occur within minutes to hours, our 24-h transcriptomic analysis was designed to capture downstream transcriptional programs rather than acute post-translational signaling events. GO and KEGG enrichment analyses indicated enrichment of metabolic pathways and epidermis-related terms (e.g., keratinization and epidermal development). Although classical MAPK and PI3K-Akt pathway genes were not significantly enriched after multiple testing correction, modest expression changes in several components were observed and are presented as hypothesis-generating supplementary information. Because RNA was extracted from full-thickness organ-cultured skin, these changes reflect bulk tissue responses and cannot be attributed solely to keratinocytes. UVB at 308 nm is absorbed predominantly in the epidermis with limited penetration into the superficial dermis, suggesting that direct UVB responses are likely epidermal, although secondary dermal responses cannot be excluded.

EL-308 enables localized delivery of high-fluence UVB to selected skin areas and is used clinically for localized inflammatory dermatoses [32,33]. Compared with whole-body phototherapy modalities, targeted irradiation may reduce exposure of surrounding uninvolved skin. However, translating ex vivo marker changes to clinical benefit requires caution, because the organ-culture model lacks systemic immune and vascular components and control tissues show degeneration over time. The tissue-level responses described here may be relevant when interpreting clinical dosing strategies, but further work in diseased skin and in vivo settings is necessary to define their clinical and safety implications.

Although transcriptomic analyses revealed promising molecular results, we did not confirm these results by qPCR or proteomic approaches. Therefore, future studies are needed to determine whether these gene-level changes translate into protein-level alterations and functional outcomes. It also remains unclear whether similar transcriptional responses occur in diseased skin, such as psoriatic or eczematous lesions, or whether lesional and non-lesional areas exhibit distinct patterns in response to EL-308.

This study also has limitations. Most notably, the number of biological replicates was restricted to three donors (*n* = 3) because the availability of human skin samples was inherently limited, which may reduce statistical power and the generalizability of the findings. Therefore, the results should be interpreted with caution. Another limitation is that immunohistochemical endpoints were assessed from 48 h onward to evaluate stable tissue-level phenotypes; thus, the lack of 24-h IHC is a limitation of the study.

Despite these limitations, the present results provide an ex vivo description of dose- and time-dependent epidermal marker changes after EL-308 exposure. Given that early time points and direct DNA-damage endpoints were not assessed, conclusions regarding net regeneration or therapeutic benefit should be made cautiously. Future studies incorporating early injury markers, diseased skin, and in vivo models will be important to clarify how targeted excimer phototherapy balances damage and repair processes.

In summary, this study characterizes tissue-level epidermal responses to 308-nm excimer light in a human organ-cultured skin model, including changes in proliferation/apoptosis labeling, differentiation markers, and accompanying transcriptional programs at 24 h after high-dose exposure. These findings should be interpreted within the limitations of the ex vivo design and limited biological replication, and they motivate further preclinical and clinical studies to define mechanisms, safety endpoints (including DNA damage), and disease-specific responses.

## 4. Materials and Methods

### 4.1. Human Organ-Cultured Skin Model

Human skin samples were obtained as surgical excess from 10 healthy adult donors (6 males, 4 females; age range: 37–89 years; median: 69 years) who underwent routine skin graft surgery. All procedures were conducted with informed consent and were approved by the Institutional Research Ethics Committee of Osaka City University (approval no. 2280), in accordance with the Declaration of Helsinki (http://www.wma.net). Anatomical sites varied depending on the surgical procedure and included the axilla, groin, back, abdomen, and face. All tissue samples were processed using a standardized protocol [34]. Human skin samples were initially obtained from 10 donors. From each donor, multiple explants were prepared and allocated to different irradiation conditions and time points. During organ culture, some explants developed culture-related tissue deterioration, including extensive necrosis, epidermal detachment, or loss of epidermal architecture, and/or did not provide sufficient matched tissue across the required conditions. These samples were therefore excluded from quantitative analysis. Quantitative immunohistochemical analyses were performed using three donors whose explants retained evaluable epidermal architecture on H&E staining and showed interpretable nuclear/cellular staining, including DAPI counterstaining where applicable, without extensive culture-related necrosis that would preclude analysis. Immediately after collection by sterile 4-mm punch biopsy (Kai industries Co., Ltd., Gifu, Japan), tissues were immersed in cold phosphate-buffered saline (PBS) and transferred to serum-free William’s E medium supplemented with 1% penicillin–streptomycin, insulin, hydrocortisone, and L-glutamine [17]. After an overnight incubation at 37 °C in a humidified 5% CO_2_ atmosphere to allow for tissue adaptation, the culture medium was replaced and samples were irradiated with EL-308 (Thera Beam UV308; USHIO, Tokyo, Japan) [35] at a dose of 300, 400, or 600 mJ/cm^2^. Fluence delivery was controlled by timer-based irradiation (manufacturer-described “timer control”). The intended fluence was set on the device and irradiation was performed with a fixed geometric condition (constant distance and perpendicular alignment between the aperture and the tissue surface), following the manufacturer’s operating procedure. The device was maintained according to the manufacturer’s recommended service/maintenance program. We did not independently measure irradiance at the sample plane using an external radiometer. A single EL-308 irradiation was performed during the culture period. For transcriptomic analysis, samples were harvested at 24 h (day 1) after the single irradiation. For histology and immunohistochemistry, samples were harvested on days 2, 5, and 7 after the single irradiation.

Collected specimens were processed for downstream applications according to their intended use. In histological and immunohistochemical analyses, each sample was fixed in 5 mL of 4% paraformaldehyde diluted in PBS and stored at 4 °C. Samples were cryopreserved for an immunofluorescence analysis, and were snap-frozen in liquid nitrogen immediately after harvest and stored at −80 °C until RNA extraction for transcriptomic analysis.

### 4.2. Immunohistochemistry

To examine the proliferation of KCs, human organ-cultured skin samples were immunostained for Ki-67 using a tyramide signaling amplification (TSA) system (Perkin Elmer, Waltham, MA, USA) according to the manufacturer’s protocol. Sections were incubated at 4 °C overnight with a rabbit anti-human Ki-67 antibody (Abcam, Cambridge, UK; 1:200 in PBS). TSA was employed to increase detection sensitivity.

To detect apoptotic cells, TUNEL staining was performed using the ApopTag Fluorescein In Situ Apoptosis Detection Kit (Millipore, Billerica, MA, USA). Sections were incubated with digoxigenin-labeled dUTP in the presence of terminal deoxynucleotidyl transferase at 37 °C for 60 min. After washing treatment with the Stop/Wash buffer, TUNEL-positive cells were visualized using a fluorescein isothiocyanate-conjugated anti-digoxigenin antibody. Ki-67 and TUNEL positivity were defined as nuclei showing specific immunoreactivity.

To detect CK10, CK16, and involucrin, deparaffinized sections were subjected to antigen retrieval by autoclaving at 121 °C for 15 min. Endogenous avidin and biotin activities were blocked at room temperature for 45 min. Sections were incubated at 4 °C overnight with the following primary antibodies diluted in PBS containing 2% goat serum: mouse anti-human CK16 (1:500; Abcam), rabbit anti-human CK10 (1:10; Abcam), and rabbit anti-human involucrin (1:500; Abcam). After washing, sections were incubated with biotinylated goat anti-rabbit or anti-mouse IgG (1:200; Jackson ImmunoResearch, West Grove, PA, USA) at room temperature for 45 min. Detection was performed using an alkaline phosphatase-based avidin–biotin complex (Vectastain Elite ABC Kit; Vector Laboratories, Burlingame, CA, USA), and expression was visualized with Fast Red substrate (Sigma-Aldrich, St. Louis, MO, USA). Nuclei were counterstained with Mayer’s hematoxylin. CK10, CK16, and involucrin positivity was defined as cytoplasmic staining within keratinocytes.

To evaluate integrin β1 expression, cryosections were permeabilized with 1% Triton X-100 at room temperature for 10 min and fixed in cold acetone (−20 °C, 2 min). Sections were incubated at 4 °C overnight with a rabbit anti-human integrin β1 antibody (Abcam, ab52971; 1:20 in PBS), followed by an incubation with a FITC-conjugated goat anti-rabbit IgG secondary antibody (Jackson ImmunoResearch) at room temperature for 45 min. Nuclei were counterstained with DAPI. Slides were mounted with Fluoromount and stored at −20 °C. Integrin β1 positivity was defined as membrane-associated fluorescence along the basal and suprabasal keratinocytes.

For each condition in each donor, multiple non-overlapping microscopic fields within the viable epidermis were examined at ×400 magnification. The epidermis was manually outlined as the region of interest (ROI) to exclude the dermis.

Ki-67- and TUNEL-positive cells were quantified as cell density, calculated as the number of positive nuclei per epidermal area (cells/mm^2^). The ROI area was calibrated using the pixel-to-distance scale in ImageJ software v1.52a (National Institutes of Health, Bethesda, MD, USA). Fluorescence intensity for integrin β1 and differentiation markers (CK10, CK16, involucrin) was quantified as the mean gray value within the epidermal ROI following standard background subtraction using the same software. No automated thresholding was applied. All images were acquired under identical exposure settings across samples and groups to ensure comparability.

Each dot in the graphs represents one microscopic field, whereas three independent donors were included in the study (*n* = 3).

For each staining, non-irradiated organ-cultured skin from the same donors was included in every experiment and served as an internal positive control, because the expected distribution of each marker in normal human epidermis is well-established (Ki-67 in basal keratinocytes, CK10 and involucrin in suprabasal layers, CK16 in suprabasal keratinocytes, and integrin β1 in basal keratinocytes). The presence of these characteristic patterns in control sections was confirmed in each staining run.

Detailed information on all antibodies, including manufacturer, host species, and catalog number, is provided in Supplementary Table S3.

### 4.3. RNA Sequencing

RNA sequencing was performed on human organ-cultured skin specimens 24 h after EL-308 irradiation at 600 mJ/cm^2^. Total RNA was extracted from irradiated and control samples using the RNeasy Mini Kit (Qiagen, Hilden, Germany) and assessed for purity (OD 260/280 = 1.8–2.0). RNA integrity was confirmed using an Agilent Bioanalyzer (Agilent Technologies, Inc., Santa Clara, CA, USA) prior to library construction. RNA libraries were constructed using the TruSeq Stranded mRNA LT Sample Prep Kit (Illumina, San Diego, CA, USA) and sequenced on an Illumina HiSeq platform with paired-end reads (2 × 100 bp). The resulting raw sequencing data have been deposited in the DNA Data Bank of Japan (DDBJ) under accession number E-GEAD-1119.

Adapter trimming and quality filtering were conducted using in-house pipelines. High-quality reads were aligned to the GRCh38 human reference genome using HISAT2 v2.1.0 [36], and transcript assembly and quantification were performed using StringTie v2.1.3b [37]. Gene expression levels were normalized using Fragments Per Kilobase of transcript per Million mapped reads (FPKM) and Transcripts Per Million.

Differential expression analysis was performed using the edgeR package (version 4.10.0) [38], with differentially expressed genes (DEGs) defined as those showing |fold change| ≥ 2 and *p* < 0.05. Functional enrichment analyses were conducted using g:Profiler [39] for GO terms and the KEGG pathway database [40].

DEGs were statistically evaluated using negative binomial models in edgeR, and *p*-values were adjusted for multiple testing using the Benjamini–Hochberg method. Genes with a false discovery rate (FDR)-adjusted *p*-value < 0.05 were considered to be significant. Regarding KEGG pathway enrichment, pathways with FDR < 0.05 were defined as significantly enriched.

The RNA-seq analysis was performed using the 600 mJ/cm^2^ condition because this dose consistently induced the strongest histological and immunohistochemical response among all irradiation conditions tested (300, 400, 600 mJ/cm^2^). Given the resource-intensive nature of RNA-seq, the experiment was designed to capture the maximal transcriptional alterations, rather than to perform a full dose–response transcriptomic analysis. Therefore, the highest effective dose (600 mJ/cm^2^) was selected to elucidate the molecular pathways underlying EL-308-induced epidermal changes. In addition, the limited availability of donor-derived human skin and ethical restrictions on its use allowed RNA-seq to be performed at only a single dose per donor, which is why multi-dose transcriptomic analysis could not be conducted.

### 4.4. Statistical Analyses

Statistical analyses for immunohistochemical quantification were performed using GraphPad Prism 8.2.1 (GraphPad Software, San Diego, CA, USA). Data are presented as the mean ± the standard deviation (SD). Comparisons among multiple groups were conducted using an ordinary one-way analysis of variance followed by Tukey’s multiple comparisons test. A *p*-value < 0.05 was considered significant. The significance of differences is indicated in the figures as follows: * *p* < 0.05, ** *p* < 0.01, *** *p* < 0.001, **** *p* < 0.0001.

## 5. Conclusions

Using a human organ-cultured skin model, the present study characterized dose- and time-dependent changes in epidermal markers following 308-nm excimer light exposure. EL-308 was associated with changes in proliferation (Ki-67), apoptosis (TUNEL), and differentiation-related markers (CK10, CK16, involucrin, and integrin β1), and RNA-seq at 24 h after 600 mJ/cm^2^ exposure indicated enrichment of metabolic pathways and epidermis-related gene sets. These findings provide descriptive tissue-level data on epidermal responses after EL-308 irradiation; however, interpretation is limited by the ex vivo design, the limited number of biological replicates, and lack of early time points and DNA-damage assessment. Further studies in diseased skin and in vivo models are needed to determine the clinical and mechanistic implications of these responses.

## Data Availability

The RNA-seq datasets generated in this study have been deposited in DDBJ under accession number E-GEAD-1119. These data will be publicly available upon publication. All other data supporting the results of this study are available from the corresponding author upon reasonable request.

## Supplementary Materials

The following supporting information can be downloaded at: https://www.mdpi.com/article/10.3390/ijms27156698/s1.
