# Supplementary material for: Direct Effects of 308-nm Excimer Light on Keratinocyte Proliferation and Differentiation in Human Organ-Cultured Skin
Source: Int J Mol Sci. 2026 Jul 27;27(15):6698. doi: 10.3390/ijms27156698 (PMC13467002; doi:10.3390/ijms27156698)
Supplement: Supplementary file 1 [file ijms-27-06698-s001.zip › Supplementary_Table_S3_Antibodies.pdf]

Supplementary Table S3. List of antibodies and staining conditions used in this study.

Marker	Host species	Dilution ratio	Manufacturer	Catalog number	Application
Ki-67	Mouse	1:10	DAKO, Agilent	M7240	IHC (paraffin)
TUNEL	-	-	Millipore	ApopTag Kit	TUNEL (paraffin)
CK10	Rabbit	1:10	Abcam	ab76318	IHC (paraffin)
Involucrin	Rabbit	1:500	Abcam	ab53112	IHC (paraffin)
CK16	Mouse	1:500	Abcam	ab8741	IHC (paraffin)
β-integrin	Rabbit	1:20	Abcam	ab52971	IF (frozen)
