# Supplementary material for: Direct Effects of 308-nm Excimer Light on Keratinocyte Proliferation and Differentiation in Human Organ-Cultured Skin
Source: Int J Mol Sci. 2026 Jul 27;27(15):6698. doi: 10.3390/ijms27156698 (PMC13467002; doi:10.3390/ijms27156698)
Supplement: Supplementary file 1 [file ijms-27-06698-s001.zip › Supplementary_Legends.pdf]

Supplementary Figure S1. KEGG pathway enrichment map of differentially expressed genes (DEGs) after EL-308 irradiation. This figure presents the results of a KEGG pathway enrichment analysis based on DEGs identified by an RNA-seq analysis of human organ-cultured skin after EL-308 irradiation. The vertical axis lists significantly enriched pathways, and the color intensity of each horizontal bar represents the significance level (adjusted p-value). Pathways are color-coded according to their functional categories. The analysis compares UVB-irradiated samples with non-irradiated controls.

Supplementary Figure S2. Non-significant changes in MAPK and PI3K-Akt pathway-associated genes 24 hours after EL-308 irradiation (600 mJ/cm^2). Fold-changes of upstream ligands (FGF2, FGF7, IGF1) and downstream effectors (ATF2, MAP4K3, RPS6KA6, PIK3CA) are shown. Although these changes did not reach statistical significance after multiple testing correction (adjusted $p \geq 0.05$), their consistent upward trend suggests a possible supportive role, but should not be interpreted as evidence of pathway activation. These data are provided as supplementary information.

Supplementary Table S1. List of differentially expressed genes (DEGs) after EL-308 irradiation. This table includes gene names, fold changes, p-values, and adjusted p-values (Benjamini-Hochberg correction). Genes with adjusted $p < 0.05$ were considered to be significant.

Supplementary Table S2. Summary of changes in the number of positive cells on immunohistochemistry in epidermis of organ-cultured human skin following EL-308 irradiation. This table provides a qualitative summary of staining results for key markers (Ki-67, TUNEL, CK10, involucrin, CK16, and integrin β1) on Day 2, 5, and 7 after EL-308 irradiation at doses of 300, 400, and 600 mJ/cm^2. Arrows (↑, ↓) indicate higher or lower expression than in non-irradiated controls. N.S. indicates no significant change.

Supplementary Table S3. List of primary antibodies used for immunostaining. The table includes information on the target antigen, host species, dilution ratio, manufacturer, catalog number, and application.
