# Supplementary material for: Direct Effects of 308-nm Excimer Light on Keratinocyte Proliferation and Differentiation in Human Organ-Cultured Skin
Source: Int J Mol Sci. 2026 Jul 27;27(15):6698. doi: 10.3390/ijms27156698 (PMC13467002; doi:10.3390/ijms27156698)
Supplement: Supplementary file 1 [file ijms-27-06698-s001.zip › Supplementary_Table_S2_Summary.pdf]

Supplementary Table S2. Summary of changes in the number of positive cells on immunohistochemistry in the epidermis of organ-cultured human skin following EL-308 irradiation.

Day	Marker	300 mJ/cm^2	400 mJ/cm^2	600 mJ/cm^2
2	Ki-67	N.S.	↑	N.S.
	TUNEL	N.S.	N.S.	N.S.
	β1-integrin	↑	↑	N.S.
	CK10	N.S.	↓	N.S.
	Involucrin	N.S.	N.S.	↑
	CK16	N.S.	↓	N.S.

Day	Marker	300 mJ/cm^2	400 mJ/cm^2	600 mJ/cm^2
5	Ki-67	N.S.	N.S.	↑
	TUNEL	N.S.	N.S.	↑
	β1-integrin	N.S.	↑	N.S.
	CK10	↓	N.S.	↓
	Involucrin	N.S.	↓	N.S.
	CK16	N.S.	N.S.	↑

Day	Marker	300 mJ/cm^2	400 mJ/cm^2	600 mJ/cm^2
7	Ki-67	N.S.	N.S.	↑
	TUNEL	N.S.	N.S.	↓
